# Corneal thickness and endothelial morphology in Normal Thai eyes

**DOI:** 10.1186/s12886-020-01385-1

**Published:** 2020-04-28

**Authors:** Napaporn Tananuvat, Natawan Khumchoo

**Affiliations:** Department of Ophthalmology, Faculty of Medicine, Chiang Mai University, Chiang Mai, 50200 Thailand

**Keywords:** Corneal endothelium, Specular microscopy, Central corneal thickness, Corneal endothelial cell density

## Abstract

**Background:**

This study aimed to determine the influence of age on central corneal thickness and corneal endothelial morphology as well as to identify the relationship between them in normal Thai eyes.

**Methods:**

Non-contact specular microscopy was performed in volunteers stratified into seven age groups ranging from 11 to 88 years. The corneal endothelial parameters studied included central corneal thickness (CCT), endothelial cell density (ECD), coefficient of variation in cell size (CV), cell area (CA) and percentage of regular hexagonal cells.

**Results:**

In a total of 501 subjects (1002 eyes), the mean age was 43.12 ± 18.80 years and 347(69.3%) were females. The mean CCT, ECD, CV, CA, and hexagonality was 533.80 ± 33.00 μm, 2732 ± 258 cell/mm^2^, 37.61 ± 6.76%, 369.04 ± 37.90 μm, and 49.03 ± 7.53%, respectively. There was a significant inverse correlation between age and CCT (r = − 0.215, *P* <  0.001), ECD (r = − 0.496, *P* <  0.001),and hexagonality (r = − 0.265, *P* <  0.001). The CV and CA directly correlated with age (r = 0.242, *P* <  0.001 and r = 0.470, *P* <  0.001). The estimate rate of endothelial cell loss was 0.2% per year. There was no correlation between CCT and ECD (*P* = 0.106).

**Conclusion:**

Normative data for corneal endothelial morphology in healthy Thai eyes showed that CCT, ECD, and hexagonality were significantly decreased, while the endothelial cell area and the variation in cell size were increased with aging. The central corneal thickness did not correlate with the endothelial cell density.

## Background

Corneal endothelial cells (CECs), originated from the neural crest, cover the posterior surface of the cornea and are made up of a monolayer of interdigitated cells arranged in a mosaic pattern of mostly hexagonal shapes [1].These metabolically active cells are responsible for regulating fluid and solute transport between the aqueous humor and corneal stromal in order to maintain normal corneal thickness and corneal transparency [2]. Unlike the corneal epithelia, the CECs cannot regenerate and decline throughout their life [2, 3].Other factors that contribute to alteration of CECs morphology include trauma [2], intraocular surgery [4], contact lens wearing [5], dry eye [6], and systemic diseases such as diabetes mellitus [7].

In order to compensate the cell loss, the surrounding CECs will be enlarged, thus wound healing is accomplished by spreading of cells to create a contiguous layer of cells on the inner surface of the cornea [2]. Apart from endothelial cell density, the coefficient of variation of the mean cell area (standard deviation of mean cell area/ mean cell area) is a clinically valuable marker and is about 0.25 in the normal cornea. This increase in the variation of cell size is termed as polymegathism. Another indication of CEC health is a percentage of hexagonality. In the normal healthy cornea, 70–80% of CECs have a hexagonal shape. This deviation from hexagonality is referred to as pleomorphism [8].

Corneal thickness is another important parameter for diagnosis of corneal disorders and treatment plan. Corneal thickness becomes more essential in determining intraocular pressure (IOP) [9]. Increased corneal thickness may give an artificially high IOP measurement, while decreased corneal thickness may give an underestimated IOP reading.

The specular microscope is a tool to assess the structure and functions of corneal endothelial cells in vivo. Currently, a non-contact specular microscope is widely used to evaluate the corneal endothelial morphology because it is non-invasive and easy to perform.

The study of corneal thickness and corneal endothelial cell morphology is needed for evaluating the cornea health. However, the results have been found to be different among various populations. Normative data are necessary for comparison between normal populations and in patients with eye diseases as well as in planning for intraocular surgery, corneal transplantation and refractive procedures, such as phakic intraocular lenses, and to study the effects of new intraocular devices or topical drugs. This study aimed to evaluate the correlation of age on central corneal thickness and corneal endothelial morphology in normal Thai eyes in different age groups, and to be used as normative data for further studies.

## Methods

This prospective cross-sectional study was based in the outpatient eye clinic, Chiang Mai University Hospital, tertiary eye care in northern Thailand, between May 2016 and March 2018. This study was approved from the Research and Ethics Committee of Faculty of Medicine, Chiang Mai University (Study code: OPT-2559-03823) and followed the tenets of Declaration of Helsinki. Written informed consent was obtained from all the participants and from a parent or guardian for participants under 16 years old after complete explanation.

Inclusion criteria were as follows: volunteers aged more than 10 years who were walk-in patients or volunteers from staffs or relatives of the patients with Thai ethnicity and nationality. Exclusion criteria included glaucoma or using anti-glaucoma medications, corneal disorders (i.e. scar, ectasia, dystrophy, and dry eye with conjunctival or corneal fluorescein staining), pterygium that involved cornea > 2 mm, recent ocular infection, previous ocular surgery or ocular trauma, history of contact lens wear, and refractive errors with spherical equivalent beyond ±6 diopters. The subjects were also excluded if having systemic conditions that may affect the cornea such as diabetes mellitus. Elderly subjects who had age-related cataract with best corrected visual acuity (VA) of ≥6/18 were recruited if the other parts of the eyes were normal.

Complete ocular examination was performed in all subjects including Snellen VA, auto-refraction, slit-lamp biomicroscopy, conjunctival and corneal fluorescein staining, fundus examination, and IOP assessment. Non-contact specular microscopy (EM4000, Tomey Corporation, Nagoya, Japan) was used to evaluate the central corneal thickness and corneal endothelium morphology. The volunteer’s head was positioned against the head band and chin rest, he or she was then instructed to look straight ahead into the fixation target. The device provided continuous capturing 16 images with the one-time operation. The best quality image was automatically selected and analysis of cell parameter was performed by the built-in software, and the results were displayed on the screen and obtained as the printout.

The main corneal parameters in this study were central corneal thickness (CCT), endothelial cell density (ECD), coefficient of variation in average cell size (CV), cell area (CA) and percentage of regular hexagonal cells (hexagonality). Volunteers were divided into seven groups of allocation by stratified age. Each group included a 10-year interval: 11 to 20, 21 to 30, 31 to 40, 41 to 50, 51 to 60, 61 to 70, and ≥ 71 years. Data analysis were performed by using the SPSS program (version 22.0, SPSS Inc., Chicago, IL, USA). The results were expressed as means and standard deviation for quantitative variables, and numbers and frequency for qualitative variables. Pearson’s correlation coefficient was used to evaluate the correlations between CCT and endothelial parameters with age. Multiple linear regressions were used to adjust the effects of gender and age with other dependent variables. Partial correlation with adjustment for age was used for analyzing the correlation between CCT and ECD. Only the data of the right eye was used for demonstrating the relationship between age, corneal endothelial parameters and CCT, while the data from the left eye was shown in supplementary materials. The parameters were compared between the right and left eye by using pair t-test because the data has shown a normal distribution. A value of *P* <  0.05 was considered statistically significant.

## Results

A total of 1002 eyes from 501 normal volunteers were studied with a mean age of 43.12 ± 18.80 (range 11–88 years). There were 347(69.3%) females and 154(30.7%) males. The mean CCT in the study population was 533.80 ± 33.00 μm. The mean ECD, CV, CA, and hexagonality were 2732.48 ± 258.51 cell/ mm^2^, 37.61 ± 6.66%, 369.04 ± 37.90 μm^2^, and 49.03 ± 7.53%, respectively (Table 1).

**Table 1:** Corneal thickness and endothelial morphology of study population in different age groups

Age (years)	Number (eyes)	CCT (μm)	CD (cell / mm^2^)	CV (%)	CA (μm^2^)	Hexagonality(%)
11–20	72	547.40 ± 36.84	2944.65 ± 231.95	34.76 ± 5.87	341.71 ± 27.02	54.06 ± 10.12
21–30	88	533.62 ± 32.07	2843.08 ± 193.29	36.25 ± 4.42	353.23 ± 23.26	50.00 ± 7.92
31–40	70	536.44 ± 28.88	2777.23 ± 206.59	37.99 ± 4.75	361.97 ± 26.93	47.74 ± 5.32
41–50	73	539.90 ± 30.24	2686.05 ± 234.18	37.56 ± 4.87	375.25 ± 34.17	47.30 ± 5.60
51–60	87	530.62 ± 32.91	2650.80 ± 178.81	38.41 ± 7.06	377.89 ± 28.91	48.40 ± 6.45
61–70	82	521.74 ± 32.70	2581.67 ± 269.58	39.77 ± 9.98	391.17 ± 48.38	48.17 ± 6.16
≥ 71	29	522.52 ± 28.26	2550.45 ± 315.72	39.45 ± 6.29	397.28 ± 50.54	45.38 ± 8.54
Total	501	533.80 ± 33.00	2732.48 ± 258.51	37.61 ± 6.66	369.04 ± 37.90	49.03 ± 7.53

*CCT* central corneal thickness, *ECD* endothelial cell density, *CV* coefficient of variation in cell size, *CA* cell area.

**Fig. 1:**
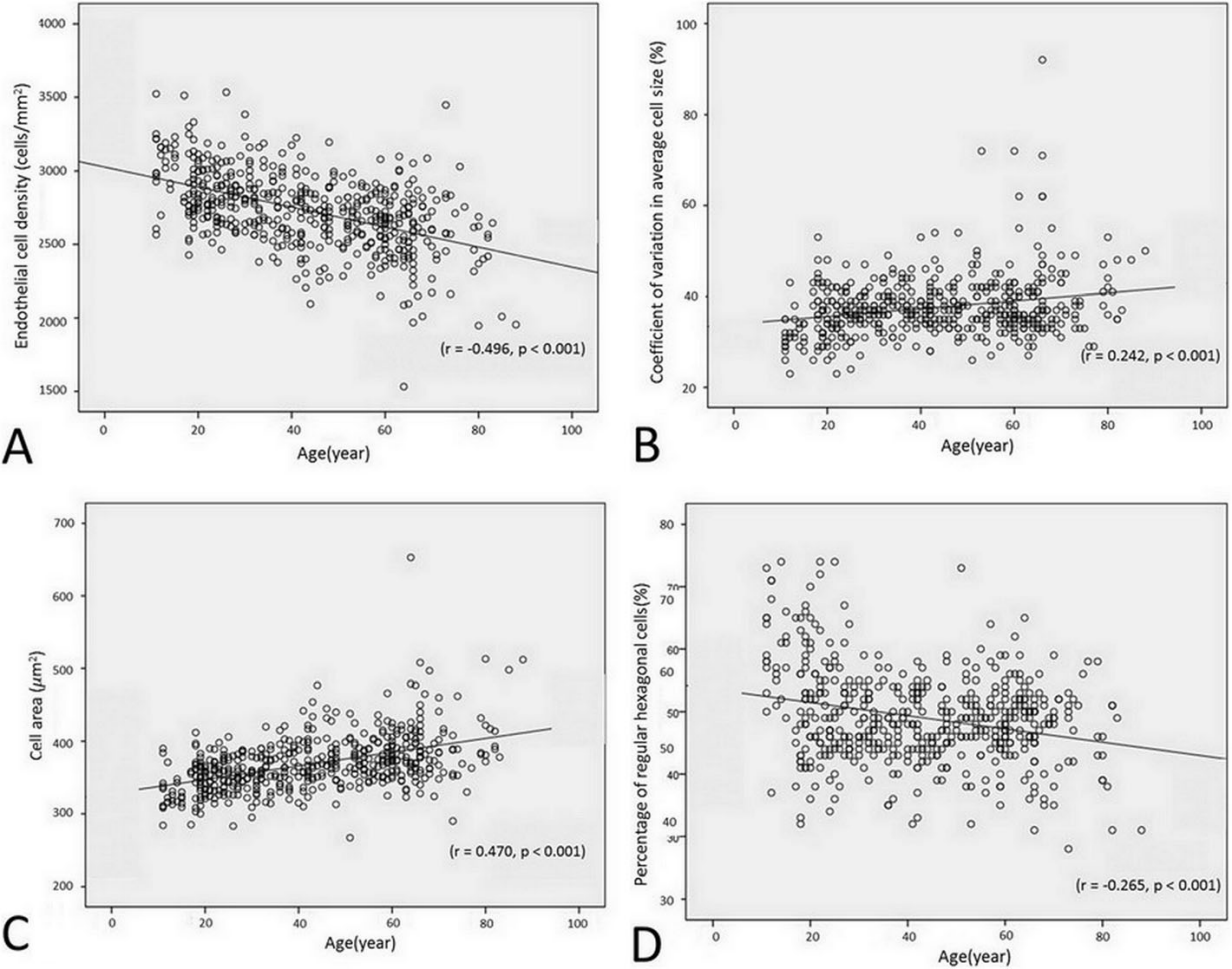
Scatter plots show the correlation between age and the endothelial cell density (A), coefficient of variation in average cell size (B), cell area (C), and percentage of regular hexagonal cells (D)

**Fig. 2:**
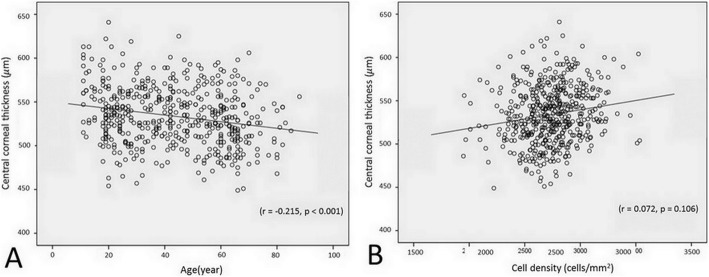
Scatter plots show the correlation between the central corneal thickness and age (A), and the endothelial cell density (B)

There were significantly inversed correlations between ECD and CCT with increased age(r = − 0.496, *p* <  0.001 and r = − 0.215, *p* <  0.001, respectively) (Figs. 1 and 2a). While the CV and CA had significantly direct correlation with increase aging (r = 0.242, *p* <  0.001 and r = 0.470, *p* <  0.001, respectively) (Fig. 1b, c). The percentage of hexagonality showed significant decrease with age (r = − 0.265, *p* <  0.001) (Fig. 1d).

When age was adjusted for the correlation between gender and corneal morphology by using multiple regression analysis, males had a significant inverse correlation with CV (ß = − 2.93, *p* <  0.001) and direct correlation with hexagonality (ß = 4.99, *P* <  0.001) (Table 2). An expected ECD for each age can be calculated using the single linear regression equation; y = a + bx.

**Table 2:** Multiple linear regression analysis for central corneal thickness and corneal endothelial parameters

	CCT		ECD		CV		CA		Hexagonality	
	ß	*p*-value	ß	*p*-value	ß	*p*-value	ß	*p*-value	ß	*p*-value
Sex (Male to Female)	− 0.043	0.989	29.973	0.172	−2.926	< 0.001	−4.021	0.219	4.999	< 0.001
Age (year)	−0.378	< 0.001	−6.722	< 0.001	0.076	< 0.001	0.934	< 0.001	−0.090	< 0.001

*ß* Beta coefficient, *CCT* central corneal thickness, *ECD* endothelial cell density, *CV* coefficient of variation in cell size, *CA* cell area.

If: y = endothelial cell density, x = age, a = 3026.53, and b = − 6.819.

By using this equation, the estimated annual endothelial cell loss rate in normal Thai eyes is 0.225%.

When the CCT and other corneal endothelial parameters were compared between eyes, only mean CCT was significantly different between the right and left eyes (Table 3). After adjusting for age, there was no correlation between the CCT and ECD (r = 0.072, *p* = 0.106) (Fig. 2b).The correlation of the left eye parameters is demonstrated in supplementary table 3 and supplementary Figs. 1−2.

**Table 3:** Comparison of the central corneal thickness and corneal endothelial morphology between eyes

Variable (mean ± SD)	Right eye (n = 501)	Left eye (*n* = 501)	p-value
CCT(μm)	533.80 ± 33.00	523.27 ± 32.43	< 0.0001
ECD(cell / mm^2^)	2732.48 ± 258.51	2730.21 ± 251.07	0.681
CV (%)	37.61 ± 6.66	37.69 ± 14.48	0.901
CA (μm^2^)	369.04 ± 37.90	369.69 ± 38.05	0.500
Hexagonality (%)	49.03 ± 7.53	48.57 ± 7.56	0.074

*CCT* central corneal thickness, *ECD* endothelial cell density, *CV* coefficient of variation in cell size, *CA* cell area.

## Discussion

This study demonstrated that the corneal endothelial cell density and the central corneal thickness decreased with aging. Although the corneal endothelial cell density and other morphology have been reported in previous studies, direct comparison of the results among studies is limited due to various evaluation methods and study populations. The results from most studies showed a trend toward decreasing cell density (Table 4) and increasing cell variation with aging. High CEC density (> 4200 cells/mm^2^) had been reported just after birth and in infants, but these values rapidly decreased during childhood and then decreased slowly after the age of 18 [21]. This evidence supports the fact that corneal endothelium lacks proliferative ability, resulting in reduction of these cells with age.

**Table 4:** Literature review of studies of corneal endothelial morphology and corneal thickness among various populations

Study	Population	N (eyes/cases)	Mean Age (range), years	Machines	Mean ECD (cell/mm)	Cell loss rate (%/year)	Correlation: ECD and Age		Mean CCT (μm)	Correlation: CCT and Age	
							r	*p* value		r	p value
Rao et al. 2000 [10]	Indian	1074/537	48 ± 16.5 (20–87)	Konan SP8000, USP(for CCT)	2525 ± 3 37	0.3	−0.387	< 0.001	533 ± 50*	–	–
Padilla et al. 2004 [11]	Filipino	640/360	53 ± 17 (20–86)	Konan P9000	2798 ± 307	–	−0.21	NA	–	–	–
Hashemian et al. 2006 [12]	Iranian	525/525 Only OD	52.7 ± 19.1 (20–80)	Topcon SP2000	1961 ± 457	0.6	−0.64	< 0.001	–	–	–
Yunliang et al. 2007 [13]	Chinese	1329/700	44 ± 21 (10–98)	Konan SP9000	2932 ± 363	0.3	−0.435	0.001	–	–	–
Niederer et al. 2007 [14]	New Zealand	85/NA	38 ± 16 (18–87)	HRT II Rostock Corneal Module	2720 ± 367	0.5	−0.615	< 0.001	555 ± 31	0.148	0.176
Higa et al. 2010 [15]	Japanese	3762/4714	59.1 ± 14.9 (> 40)	Topcon SP2000	2943 ± 387	0.25	− 0.34	< 0.001	NA	–	–
Mohammad-Salih et al. 2011 [16]	Malay	125/125	45.8 ± 20.7 (20–87)	Topcon SP3000	2648 ± 310	–	− 0.300	0.001	–	–	–
Galgauska et al. 2013 [17]	Lithuania	358/211	NA (20–89)	Konan SP9000	2931 ± 371 ** to 2222 ± 182 ***	–	−0.650	0.01	563 ± 44* to 540 ± 35**	−0.156	0.01
Arici et al. 2014 [18]	Turkish	252/126	44.3 ± 13.5 (20–70)	Topcon SP3000	2732 ± 305	1.9–5.9	−0.388	< 0.001	521 ± 33	− 0.241	< 0.001
Ewete et al. 2016 [19]	Nigerian	359/201	50.35 ± 20.13 (20–93)	Konan SP9000	2610 ± 371	–	−0.318	< 0.001	–	–	–
Abdellah et al. 2019 [20]	Egyptian	568/568	49 ± 15.2 (20–85)	Topcon SP-IP	2647 ± 387	0.3	−0.357	< 0.001	514 ± 43	− 0.133	0.007
This study	Thai	1002/501 Only OD	43.12 ± 18.80 (11–88)	Tomey EM4000	2732 ± 258	0.225	−0.484	< 0.001	533 ± 33	− 0.212	< 0.001

*ECD* endothelial cell density, *CCT* central corneal thickness, *USP* ultrasound pachymeter

*CCT decreased with age but not statistically significant, **age 20–29 years old, ***age 80–89 years old

**Table 5:** Corneal thickness and endothelial morphology of the left eyes of study population in different age groups

Age (years)	Number (eyes)	CCT (μm)	CD (cell/ mm^2^)	CV (%)	CA (μm^2^)	Hexagonality(%)
11–20	72	535.61 ± 34.92	2935.82 ± 215.37	34.47 ± 5.07	342.39 ± 25.05	53.44 ± 10.21
21–30	88	522.63 ± 31.72	2830.72 ± 186.37	35.93 ± 4.21	354.75 ± 23.02	49.93 ± 7.31
31–40	70	526.99 ± 30.02	2771.76 ± 212.86	36.86 ± 4.43	362.87 ± 27.95	47.54 ± 6.60
41–50	73	528.88 ± 28.57	2687.21 ± 231.40	37.73 ± 4.40	375.04 ± 34.56	46.40 ± 5.85
51–60	87	520.09 ± 32.05	2645.55 ± 174.81	40.83 ± 32.53	378.55 ± 27.92	47.89 ± 6.57
61–70	82	513.06 ± 33.64	2601.68 ± 257.91	38.98 ± 7.90	389.70 ± 43.79	46.99 ± 6.35
≥ 71	29	509.90 ± 27.91	2540.17 ± 326.92	39.83 ± 4.38	402.66 ± 65.79	46.90 ± 7.23
Total	501	523.27 ± 32.43	2730.21 ± 251.07	37.69 ± 14.48	369.69 ± 38.05	48.57 ± 7.56

*CCT* central corneal thickness, *ECD* endothelial cell density,

*CV* coefficient of variation in cell size, *CA* cell area

The mean ECD in this study was 2732 ± 258 cell/mm^2^, which was similar to the results from other studies using non-contact specular microscope in normal eyes (Table 4). These included Filipino (2798 ± 307) [11], Malay (2648 ± 310) [16], Turkish (2732 ± 307) [18], Egyptian (2647 ± 387) [20], Nigerian (2610 ± 371) [19], and Indian (2525 ± 337) [10]. The mean ECD in Thai eyes was lower than those of Japanese (2943 ± 387) [15] and Chinese (2932 ± 363) [13], but higher than the results of Iranian (1961 ± 457) [12].The corneal diameter has been postulated to responsible for the variation of ECD in various populations as the corneal diameter might be inversely proportional to endothelial cell density as the Indian and American had less ECD than the Japanese people [10, 22]. However, one study using the confocal microscopy and the Orbscan corneal topography did not find the correlation between ECD and corneal diameter in the elderly eyes [23].The different results among studies could be due to the different specular microscope used in each study as well.

The annual endothelial cell loss rate in this study was 0.23%, which was similar to the results in Chinese (0.3%) [13], Indian (0.3%) [10], Egyptian (0.3%) [20], and Japanese (0.25%) [15], while this was lower than those from the Middle East and Caucasians i.e. Iran (0.6%) [12]. Yunliang et al. reported the annual cell loss of 0.3% in a normal Chinese population [13]. However, they noted a variation in the cell loss rate in different age groups with a higher loss of cells in the younger age groups. They also suggested that an exponential function to determine the rate of cell loss might be more appropriate than using linear regression analysis. Niederer et al. used in vivo confocal microscopy with contact method for studying corneal morphology and found that the annual rate of CEC cell loss was 0.5% [14].

Aging also influenced other corneal parameters. This study found that age had a direct correlation with the variation of cell size and cell area, and had an inverse correlation with hexagonality. The negative impacts of age on the cell variation (Indian, Chinese, Malay, Filipino) [10, 11, 13, 16], cell size (Malay, Chinese, Indian, Iranian, Turkish, Filipino) [10, 11, 13, 18], and cell shape (Indian, Chinese, Turkish) [10, 13, 18] were previously reported from different study populations. One study in Lithuania did not show the correlation of age on the CV and hexagonality [17].

For the influence of gender on CECs, after adjustment for age this study found that males had a significant inverse correlation with CV and direct correlation with hexagonality. This means that CECs in males are supposed to have fewer variables in size and had more hexagonal shape than in females. However, there was no difference of the ECD among genders. Another study reported that men’s corneal endothelial cells are more regular and have more hexagonal cells [24].These findings were different from studies in the Filipino [11] and Japanese population [15], in which women had significantly greater ECD than that in men. Some studies including Malay [16], Turkish [18], Iranian [12], and Egyptian [20] found no significant differences of mean ECD between genders.The disagreement between studies indicates that the influence of sex on corneal endothelial cells still requires more studies to justify the findings.

Corneal thickness, another important indicator of corneal health and changes in corneal endothelial function, becomes more important in determining the IOP and in planning for refractive surgery. In general, the cornea becomes edematous if the CECs decrease or loss of function. This study demonstrated that the CCT decreased with increasing age and this was similar to studies in Lithuania [17], Turkish [18], and Egyptian [20] populations (Table 4). The decrease CCT with aging may be due to the degenerative changes in corneal structures such as the thinning of corneal stroma, nerve, epithelium, or the CEC body which needs further study to investigate the answer.

This present study found that there was no correlation between CCT and the ECD after adjustment for age. Müller et al. investigated the ECD and corneal thickness in different areas of the cornea in elderly eyes using the confocal microscopy and corneal topography (Orbscan II). They found that ECD significantly correlated with CCT and corneal curvature [23].They suggested that in an older population, low ECD values would be expected in thinner and /or steeper cornea. Results from a population-based study in Japan, using the ultrasound pachymeter (USP) for evaluating the CCT in adult volunteers (age more than 40), found that CCT significantly correlated with ECD even though they suggested that the results may not be clinically significant (r = 0.071) [15]. Although the USP is a frequently used pachymeter, the main disadvantage of this device is the variation among examiners due to the inaccuracy of the probe alignment causing errors in measurement [25]. Our study showed that there was a significantly difference of CCT between the right and left eye, which might be caused by the measurement errors such as the eye’s misalignment. Previous studies found that CCT varies over the day (circadian CCT) as the cornea is thicker in the morning and gradually become thinner. This may reflect the change in corneal metabolism occurring during the night with the increase lactate and corneal swelling. To reduce this error, CCT and ECD should be measured at the same time of the day [26, 27]. In addition, the CCT measurement may be affected if performs after applanation tonometry, even though previous study found no significant influence [28]. Nevertheless, the difference in CCT between eyes found in this study (10 μm) may not be clinically significant.

This study used the non-contact specular microscopy that could assess both the corneal endothelial morphology and the central corneal thickness. This non-contact device has advantages of reducing the risk of corneal epithelial injury, transmission of infection, artifacts resulting from corneal manipulation and also providing comfort for the volunteers. However, there were some limitations of this study. First; the ECD in different areas of the cornea were not investigated as well as the relationship of corneal ECD and other parameters such as corneal diameter and curvature, axial length, anterior chamber depth, and refractive errors. Second; this study may be confounded by mild degree and asymptomatic dry eye subjects. As previous study found that corneal ECD significantly decreased in dry eye patients and correlated with clinical severity [6]. The possible mechanisms for endothelial cell loss supposed to be due to the reduced corneal nerve and the associated inflammation in dry eye disease [6, 29]. Nonetheless, the authors noted that ECD might not be affected in mild cases of dry eye which are commonly encountered in clinical practice [6].Third; this study did not adjust the effect of IOP on CCT. Last; there may be some other potential confounding factors such as smoking or nutrition. Therefore, further prospective longitudinal studies are required to evaluate their effects on the change of corneal endothelial cells with aging.

## Conclusion

This study on corneal endothelial morphology in the Thai population showed that central corneal thickness, endothelial cell density, and hexagonality were significantly decreased, while cell size and cell variation were increased with aging. The central corneal thickness did not correlate with the endothelial cell density. Ultimately, the results of this study can be used as normative data for further studies.

## Supplementary information

**Additional file 1: Suppl Fig. 1.** Scatter plots show the correlation between age and the endothelial cell density (A), coefficient of variation in average cell size (B), cell area (C), and percentage of regular hexagonal cells (D) of the left eyes.

**Additional file 2: Suppl Fig. 2.** Scatter plots show the correlation between the central corneal thickness and age (A), and the endothelial cell density (B) of the left eyes.

**Additional file 3: Table 5.** Supplementary table.

## Data Availability

The datasets used and/or analyzed during the current study available from the corresponding author on reasonable request.

## Abbreviations

CA: Cell area
CCT: Central corneal thickness
CECs: Corneal endothelial cells
CV: Coefficient of variation in cell size
ECD: Endothelial cell density
IOP: Intraocular pressure
USP: Ultrasound pachymeter
VA: Visual acuity
