# Supplementary figures and images for: Corneal thickness and endothelial morphology in Normal Thai eyes
Source: BMC Ophthalmol. 2020 Apr 28;20:167. doi: 10.1186/s12886-020-01385-1 (PMC7187506; doi:10.1186/s12886-020-01385-1)

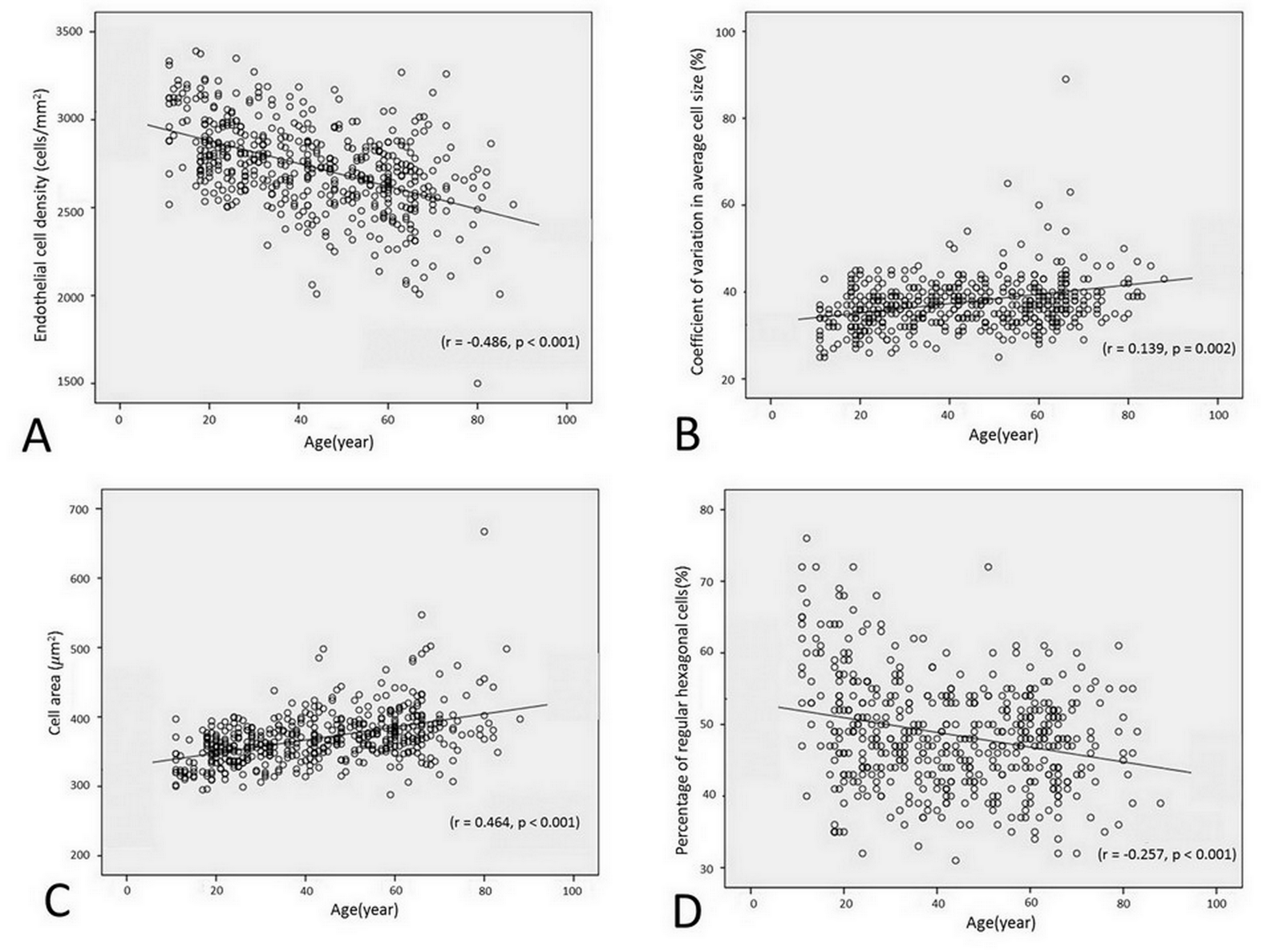

Supplement: Supplementary file 1 — Additional file 1: Suppl Fig. 1. Scatter plots show the correlation between age and the endothelial cell density (A), coefficient of variation in average cell size (B), cell area (C), and percentage of regular hexagonal cells (D) of the left eyes. [file 12886_2020_1385_MOESM1_ESM.jpg]

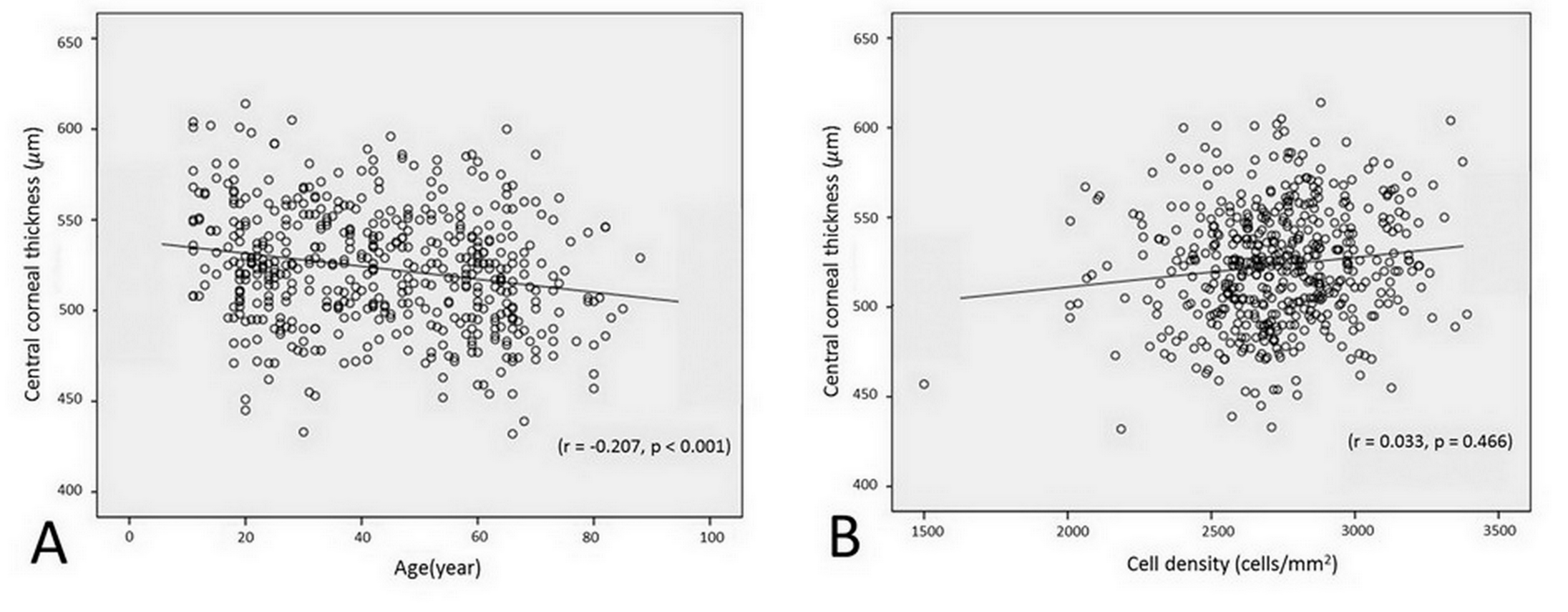

Supplement: Supplementary file 2 — Additional file 2: Suppl Fig. 2. Scatter plots show the correlation between the central corneal thickness and age (A), and the endothelial cell density (B) of the left eyes. [file 12886_2020_1385_MOESM2_ESM.jpg]
