# Supplementary material for: Corneal thickness and endothelial morphology in Normal Thai eyes
Source: BMC Ophthalmol. 2020 Apr 28;20:167. doi: 10.1186/s12886-020-01385-1 (PMC7187506; doi:10.1186/s12886-020-01385-1)
Supplement: Supplementary file 3 — Additional file 3: Table 5. Supplementary table. [file 12886_2020_1385_MOESM3_ESM.docx]

| Age (years) | Number (eyes) | CCT  (μm) | CD  (cell/ mm^2^) | CV  (%) | CA  (μm^2^) | Hexagonality ( %) |
| --- | --- | --- | --- | --- | --- | --- |
| 11-20 | 72 | 535.61±34.92 | 2935.82±215.37 | 34.47±5.07 | 342.39±25.05 | 53.44±10.21 |
| 21-30 | 88 | 522.63±31.72 | 2830.72±186.37 | 35.93±4.21 | 354.75±23.02 | 49.93±7.31 |
| 31-40 | 70 | 526.99±30.02 | 2771.76±212.86 | 36.86±4.43 | 362.87±27.95 | 47.54±6.60 |
| 41-50 | 73 | 528.88±28.57 | 2687.21±231.40 | 37.73±4.40 | 375.04±34.56 | 46.40±5.85 |
| 51-60 | 87 | 520.09±32.05 | 2645.55±174.81 | 40.83±32.53 | 378.55±27.92 | 47.89±6.57 |
| 61-70 | 82 | 513.06±33.64 | 2601.68±257.91 | 38.98±7.90 | 389.70±43.79 | 46.99±6.35 |
| ≥ 71 | 29 | 509.90±27.91 | 2540.17±326.92 | 39.83±4.38 | 402.66±65.79 | 46.90±7.23 |
| Total | 501 | 523.27±32.43 | 2730.21±251.07 | 37.69±14.48 | 369.69±38.05 | 48.57±7.56 |

**Table 5: Corneal thickness and endothelial morphology of the left eyes of study population in different age groups**

CCT: central corneal thickness, ECD: endothelial cell density,

CV: coefficient of variation in cell size, CA: cell area
